# Proximal Corpus Cavernosum Tear Presenting as Scrotal Hematoma

**DOI:** 10.1155/2013/482098

**Published:** 2013-05-23

**Authors:** A. Darves-Bornoz, M. M. DeBartolo, A. M. Mishail

**Affiliations:** Department of Urology, Stony Brook Medicine, Stony Brook, NY 11794, USA

## Abstract

We present the case of a penile fracture involving the proximal corpus cavernosum, presenting as a hematoma extending into the scrotum. The presentation, diagnosis, and surgical approach, which differ from the more typical penile shaft fracture, are delineated.

## 1. Case Presentation

A 47-year-old male presented with right hemiscrotal swelling and pain, one week following painful sexual intercourse. Over the course of the week, his pain worsened and scrotal swelling progressed, after which he presented to the Emergency Department for evaluation. Patient denied gross hematuria. He continued to have painful erections since symptom onset and denied any obstructive or irritative voiding symptoms. His medical, surgical, family, and social history were unremarkable. 

Physical examination was significant for a right scrotal induration and tenderness extending deep into the perineum sparing both testicles. Urinalysis was unremarkable. Initially, scrotal ultrasound revealed a complex cystic structure in the right scrotal wall. This study did not establish a definitive diagnosis. Subsequently, scrotal MRI was performed, which revealed a focal tear of the tunica albuginea overlying the right proximal corpus cavernosum. Associated with this was a 1 cm intracavernosal hematoma, as well as a 4 cm hematoma tracking anteriorly through the perineum toward the right hemiscrotum.

Because of these MRI findings, the patient was sent to the operating room. The deep scrotal and perineal tissues were explored through a midline perineal incision, where a large hematoma was encountered contained within ischiocavernosus muscle. The muscle was incised and dissection was carried out to the right corporeal body in order to evacuate the hematoma. After multiple rounds of copious irrigation, hemostasis was achieved and a right 1 cm oblique corporeal tear was visualized. This was repaired primarily in a watertight fashion with interrupted figure-of-eight polydioxanone sutures (PDS). The incision was then closed in layers. We left a penrose drain in the hematoma space and delivered it through a separate perineal stab wound incision.

## 2. Discussion

Penile fracture is an underreported condition in which trauma to the erect penis, most often in the setting of sexual intercourse, tears the tunica albuginea and corpus cavernosum to produce a clinically evident hematoma that deforms and detumesces the erect penis. Because of proximity to the vulnerable spongy urethra, the urethra is secondarily involved in approximately 14.2% of cases [1]. The usual insult is a “head on” trauma from the erect penis striking the pubic symphysis or perineum, almost invariably tearing the distal corpus cavernosum. Indeed, out of over one thousand previously reported penile fracture cases, only in one other case [2] was the corporeal defect found in the proximal corpus cavernosum. Thus we offer, to the best of our knowledge, the second reported case of proximal corporal cavernosal fracture. 

As was true with the previous proximal penile fracture case reported by Pruthi et al., presentation was less acute and more insidious in nature, as that case had presented with increasing penile and perineal pain and “butterfly” perineal ecchymosis over the week following the inciting event. In our case as in the previous case, the diagnosis was not clinically evident but required ancillary imaging studies for confirmation and differentiation from other clinical entities. Unlike the previous case, however, our patient presented with a scrotal hematoma that arose by tracking from the proximal corporeal body. The surgical approaches were similar: exposing the proximal corporeal body, evacuating the hematoma, and primarily repairing the corporeal tear through a midline perineal incision. This differs markedly from the standard approach to more typical cases of distal penile fracture, which exposes the defect through a circumferential degloving incision [3]. 

## 3. Conclusion 

Though this case of proximal penile fracture represents only the second reported case of its kind, its implications and applicability are more widespread given the under-reported nature of penile trauma. The clinical ambiguity of this diagnosis places patients at risk not receiving definitive surgical therapy, which confers a 30% risk of penile curvature, missed urethral injuries, and other complications [3]. As in most other penile fracture cases, the history of present illness may be vague and incomplete. High index of suspicion and choosing appropriate imaging modality is crucial in diagnosing proximal penile fracture. This avoids delay in surgical treatment and complications of a missed penile fracture. 
